# Tricyclic Antidepressant Structure-Related Alterations in Calcium-Dependent Inhibition and Open-Channel Block of NMDA Receptors

**DOI:** 10.3389/fphar.2021.815368

**Published:** 2022-02-14

**Authors:** Yulia D. Stepanenko, Dmitry A. Sibarov, Natalia N. Shestakova, Sergei M. Antonov

**Affiliations:** Sechenov Institute of Evolutionary Physiology and Biochemistry of the Russian Academy of Sciences, Saint-Petersburg, Russia

**Keywords:** NMDA receptor, tricyclic antidepressants, amitriptyline, desipramine, clomipramine, ion channel, calcium

## Abstract

*N*-methyl-D-aspartate receptors (NMDARs) are an essential target for the analgetic action of tricyclic antidepressants (TCAs). Their therapeutic blood concentrations achieve 0.5–1.5 μM, which, however, are insufficient to cause *in vitro* the open-channel block known as the only effect of TCAs on NMDARs. Whereas structures of amitriptyline (ATL), desipramine (DES), and clomipramine (CLO) are rather similar these compounds manifest different therapeutic profiles and side effects. To study structure-activity relationships of DES and CLO on NMDARs, we measured IC_50_s as a function of extracellular calcium ([Ca^2+^]) and membrane voltage (V_m_) of NMDAR currents recorded in cortical neurons. Here two components of TCA action on NMDARs are described, which could be characterized as the Ca^2+^-dependent inhibition and the open-channel block. DES demonstrated a profound Ca^2+^-dependent inhibition of NMDARs, while the CLO effect was weak. DES IC_50_ exhibited an e-fold change with a [Ca^2+^] shift of 0.59 mM, which is consistent with ATL. The Ca^2+^ dependence of NMDAR inhibition by DES disappeared in BAPTA loaded neurons, suggesting that Ca^2+^ acts from the inside. Since CLO differs from DES and ATL by the presence of Cl-atom in the structure, most likely, this is the atom which is responsible for the loss of pronounced [Ca^2+^] dependence. As for the NMDAR open-channel block, both DES and CLO were about 5-folds more potent than ATL due to their slow rates of dissociation either from open and closed states. DES demonstrated stronger V_m_-dependence than CLO, suggesting a deeper location of the DES binding site within the ion pore. Because DES and CLO differ from ATL by the nitrogen-containing tricycle, presumably this moiety of the molecules determines their high-affinity binding with the NMDAR channel, while the aliphatic chain mono-methyl amino-group of DES allows a deep permeation in the channel. Thus, different structure-activity relationships of the Ca^2+^-dependent inhibition and V_m_-dependent open-channel block of NMDARs by DES and CLO suggest that these processes are independent and most likely may represent an action on different molecular targets. The proposed model of TCA action on NMDARs predicts well the experimental values of IC_50_s at physiological [Ca^2+^] and within a wide range of V_m_s.

## Introduction

**FIGURE 1:**
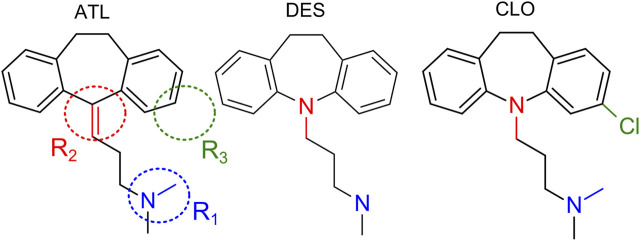
Structures of amitryptiline (ATL), desipramine (DES) and clomipramine (CLO). R_1_, R_2_, R_3_ depict regions of chemical structures which are of interest with respect to their structure-activity relationships.

Tricyclic antidepressants (TCAs, Figure 1) are widely utilized for the therapy of depression (for review Gillman 2007), neuropathic pain, and itch (for review Belinskaia et al., 2019). The pool of clinically used TCAs demonstrate numerous different molecular targets and cause inhibition of monoamine uptake, M-cholinolytic, antihistamine, and α-adrenolytic activities (for review Belinskaia et al., 2019), block of Na^+^, K^+^ and Ca^2+^ channels (Lavoie et al., 1990; Pancrazio et al., 1998; Zahradník et al., 2008; Lawson 2017). Some TCAs like amitryptiline (ATL) also inhibit Na^+^/Ca^2+^ exchange in synaptosomes (Lavoie et al., 1990).

It has been demonstrated recently that nerve injury potentiates *N*-methyl-D-aspartate receptor (NMDAR) activity at both pre- and postsynaptic sites suggesting that excitatory glutamatergic synaptic transmission is involved in the sensitization of neuropathic pain (Chen et al., 2014; Li et al., 2016). The use of antidepressants as adjuvant therapy for ameliorating chronic pain is a promising treatment strategy for patients displaying both neuropathic pain and depression (for review Dharmshaktu et al., 2012). Among antidepressants with analgesic properties, ATL has long been successfully used to treat pain (Bryson and Wilde 1996). These observations make it possible to consider inhibitors of NMDARs as perspective compounds against neuropathic pain (Peterson et al., 2021). Indeed, TCAs, including ATL and desipramine (DES), perform the open-channel block of NMDARs (Tohda et al., 1995; Barygin et al., 2017; Stepanenko et al., 2019). The efficacy of ATL, DES, clomipramine (CLO), and other TCAs against neuropathic pain is not associated with the inhibition of monoamine uptake (for review Gillman 2007) but depends on inhibition of NMDARs (Eisenach and Gebhart 1995). In addition, DES exhibits antidepressant properties inhibiting NMDARs at clinically relevant micromolar concentrations measured in human blood plasma (Kiss et al., 2012; Santos et al., 2017).

Whereas the role of NMDAR as a target for the therapeutic action of TCAs in the neuropathic pain appears to be well proved, the concentrations of ATL (Barygin et al., 2017; Stepanenko et al., 2019) and DES (Reynolds and Miller 1988; Sernagor et al., 1989) required to evoke the open-channel block of NMDARs are far above the clinically relevant values obtained in the blood plasma during the therapy (Santos et al., 2017). To overcome this contradiction, one may suggest an existence of different mechanisms of TCA-inhibition of NMDARs. In agreement, we have recently demonstrated that regardless of the open-channel block, ATL additionally induces a calcium-dependent and voltage-resistant inhibition of NMDARs since the increase of calcium concentration in the extracellular solution substantially enforces the NMDAR inhibition by ATL (Stepanenko et al., 2019). Some similar effects on NMDARs could be achieved by ethanol (Boikov et al., 2020) and lithium (Sibarov et al., 2018) or by an inhibitor (KB-R7943) of Na^+^/Ca^2+^-exchangers (Sibarov et al., 2015), which all as known can provoke the calcium-dependent desensitization of NMDARs (Sibarov et al., 2015). These agents and extraction of cholesterol from the plasma membrane of neurons, which presumably may cause an uncoupling of a tight interaction between Na^+^/Ca^2+^ exchangers, and NMDARs in the plasma membrane lipid rafts (Sibarov et al., 2018), increase the intracellular pre-membrane calcium concentration and enhance the calcium-dependent desensitization of NMDAR. Taking into account possible ATL action on Na^+^/Ca^2+^ exchanges (Lavoie et al., 1990), a similar mechanism might be involved in the Ca^2+^-dependence of NMDAR inhibition by ATL (Stepanenko et al., 2019).

The calcium-dependent voltage-resistant NMDAR inhibition has not been yet shown for DES and CLO (Figure 1). While these compounds and ATL have very close structures and, in general, differ by the aliphatic chain side-amino groups, which are -NH(CH_3_)_2_ for ATL and CLO, and -NH_2_CH_3_ for DES, by tricyclic groups which have N-atom in DES and CLO instead of C-atom in ATL, and, finally, an additional Cl-atom is present in the structure of CLO, these three TCAs manifest very different therapeutic profiles and side effects (Gillman 2007; Riediger et al., 2017). To provide more clues for structural determinants of different types of TCA action on NMDARs, here we investigate the structure-activity relationships of calcium-dependent inhibition and open-channel block of NMDARs by DES and CLO. With this goal, effects of different concentrations of DES and CLO as a function of extracellular calcium concentration ([Ca^2+^]) and membrane voltage (V_m_) were studied on NMDA elicited whole-cell currents recorded in cortical neurons. These neurons in primary culture express NMDARs of GluN1/GluN2A and GluN1/GluN2B subunit compositions (Monyer et al., 1994; Paoletti et al., 2013). The data for ATL were recruited for a comparison (Stepanenko et al., 2019). We demonstrate that NMDAR calcium-dependent inhibition and open-channel block have different dependence on the TCA structure. The observations presented here favor the independent origin of these two mechanisms of TCA action on NMDARs.

## Methods

### Primary Culture of Cortical Neurons

All procedures on animals were performed in accordance with the guide of the Federation for Laboratory Animal Science Associations (FELASA) and were approved by the Animal Care and Use Committees of Sechenov Institute. Briefly, 17 days pregnant Wistar rats (supplied by the Sechenov Institute Animal Facility) were sacrificed by 1 min CO_2_ inhalation in a plastic box connected to a CO_2_ tank. Fetuses were removed, and then primary cultures of rat cortical neurons were prepared using conventional procedures as described earlier (Antonov and Johnson 1996; Mironova et al., 2007). Neurons were grown in Neurobasal culture medium supplemented with B-27 (Gibco-Invitrogen, United Kingdom) on 7 mm glass coverslips coated with poly-D-lysine and were used for experiments after 10–14 days in culture (Mironova et al., 2007; Han and Stevens 2009).

### Patch Clamp Recordings

Whole-cell currents were recorded from cultured rat cortical neurons using a MultiClamp 700B patch-clamp amplifier. Recordings were low-pass filtered at 400 Hz and digitized at the acquisition rate of 20,000 samples per second by Digidata 1440A controlled by pClamp v10.6 software (Molecular Devices). Solution exchange was performed by means of a fast solution application system as described earlier (Sibarov et al., 2015). Unless otherwise specified, the external bathing solution contained (in mM): 144 NaCl; 2.8 KCl; 1.0 CaCl_2_; 10 HEPES, at pH 7.2–7.4, osmolarity 310 mOsm. The intrapipette solution contained (in mM): 120 CsF, 10 CsCl, 10 EGTA, and 10 HEPES, osmolarity 300 mOsm, with pH adjusted to 7.4 with CsOH. Patch pipettes of 4–6 MΩ were pulled from Sutter BF150-89-10 borosilicate capillaries. Experiments were performed at 22–25°C. Under control conditions, neurons were voltage clamped at −70 mV. Data are reported without corrections for liquid junction potential, which was measured as −11 mV. To activate NMDARs, 100 µM NMDA was always co-applied with 10 µM glycine as co-agonist. Some experiments were performed on neurons loaded with BAPTA. To load BAPTA, cortical neuron cultures were incubated in a bathing solution containing 5 µM BAPTA-AM for 1 h.

### Drugs

Compounds were acquired from Sigma-Aldrich, St. Louis, MO, United States.

### Analysis of Membrane Currents

To determine the blocking potency of TCAs, NMDA elicited currents were measured in the absence and presence of different TCA concentrations ([B]). Amplitudes of currents measured in the presence of blocker (I_b_) were normalized to maximal current response in control (I_c_). The IC_50_ is the concentration of TCA that cause 50% of the maximal inhibition of currents, and the Hill coefficient (*h*) were estimated by fitting concentration-inhibition curves with the Hill equation:

$${\text{I}}_{\text{b}}/{Ι}_{c}=1/\left(\mathrm{1+}{\left[Β\right]}^{h}\mathrm{/Ι}{\text{C}}_{50}^{\text{h}}\right)$$ (1)

NMDAR current relaxations during block or unblock by TCA were fit with the single exponential equation:

$$\text{I }=\left({\text{I}}_{\text{max}}{-\text{I}}_{\text{min}}\right)\text{·exp }\left(-\text{t/τ}\right){+\text{I}}_{\text{min}}$$ (2)

where I_max_ and I_min_ are maximal and minimal amplitudes of currents during relaxations and τ represents the time constant of the exponential component during block onset (τ_on_) or offset (unblock, τ_off_). From these measurements, the rate constants of block (k^+^) and unblock (k^−^) was estimated. k^+^ was obtained from τ_on_ measured at 20 and 90 μM DES (C_1_ and C_2_ respectively) using the equation:

$${\text{k}}^{+}=\left({1\text{ /τ}}_{\text{onC}2}{-1\text{/τ}}_{\text{onC}1}\right)\text{/}\left({\text{C}}_{2}{\;-\text{ C}}_{1}\right)$$ (3)

The k^−^ was the reciprocal to measured τ_off_:

$${\text{k}}^{-}{=1\text{ /τ}}_{\text{off}}$$ (4)

The equilibrium dissociation constant (K_d_) for DES was calculated as follows:

$${\text{K}}_{\text{d}}{=\text{k}}^{-}{\text{/ k}}^{+}$$ (5)

To obtain the voltage-dependence of NMDAR block by TCAs, the concentration-ihibition curves were generated at three membrane holding potentials V_m_ = −100, −70, and −30 mV. The obtained values were fitted with the modified by incorporating the dependence of NMDAR inhibition on [Ca^2+^] (Stepanenko et al., 2019) Woodhull equation:

$${\text{IC}}_{50}\left({\text{V}}_{\text{m}}\left[{\text{Ca}}^{2+}\right]\right){=\text{IC}}_{50}\left(\text{0 mV}\right)\text{·exp}\left({\text{V}}_{\text{m}}\text{zδF/RT}\right)\text{·exp}\left(-\left[{\text{Ca}}^{2+}\right]\text{/X}\right)$$ (6)

where IC_50_ (0 mV) and δ were set as free parameters. IC_50_ (0 mV) is the value of IC_50_ at V_m_ = 0 mV, δ is the fraction of the membrane voltage field exerting force on TCA at its binding site. z = 1 is a molecule electric charge for both DES and CLO. For Faraday constant, gas constant, and room temperature of 28°C, the RT/F ≈ 26 mV. RT/(zδF) is equal to V_m_, producing an e-fold change of IC_50_, and X is equal to [Ca^2+^], producing an e-fold change of IC_50_.

The rise time of currents activated by the testing NMDA + Gly application in trapping channel block experiments was fitted in ClampFit (pClamp, Axon Instruments) using the double exponential function:

$${\text{I}=\text{I}}_{1}\text{·exp}\left({-\text{t/τ}}_{1}\right){+\text{I}}_{2}\text{·exp}\left({-\text{t/τ}}_{2}\right)-\text{ C}$$ (7)

where I_1_ and I_2_ are amplitudes of the exponential and τ_1_ and τ_2_ are time constants of the exponential and C equals I_1_ + I_2_ + I_b_.

### Statistical Analysis

Data are presented as representative measurements as well as mean values ± standard error of the mean (SEM). Sample number (*n*) refers to the number of recorded cells. Groups were compared using ANOVA with Bonferroni correction and Student’s two-tailed *t*-test. Statistical significance is reported in the figures according to the following symbols *** and ****, which indicate *p* values below (<) 0.001 and 0.0001, respectively. Curve fitting was performed using OriginPro software (OriginLab Corp.). IC_50_s obtained from individual experiments performed in the same experimental conditions were averaged to get mean ± SEM values.

## Results

### An Extent of NMDAR Ca^2+^-dependent Inhibition Induced by Desipramine and Clomipramine Differs

**FIGURE 2:**
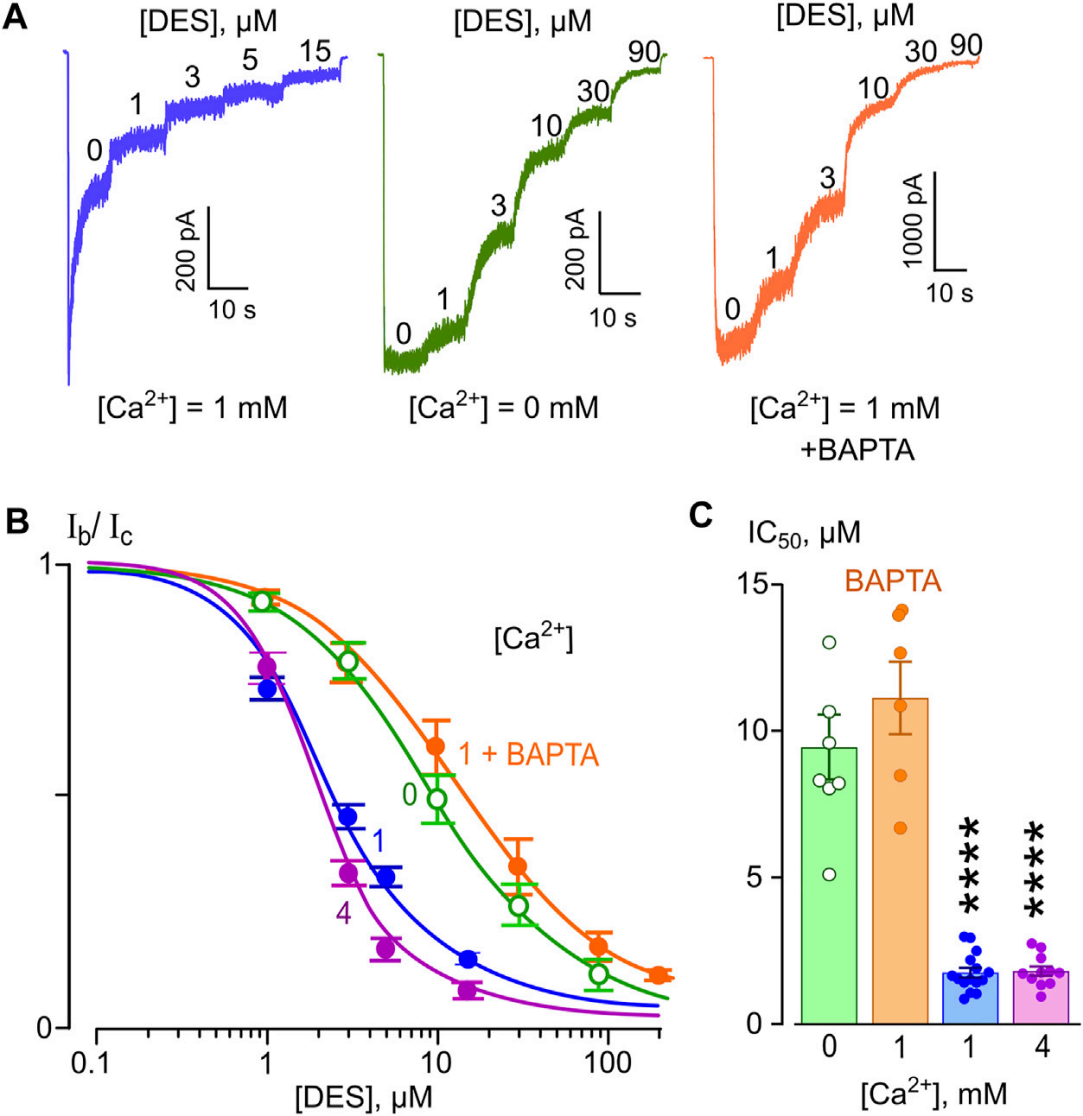
Ca^2+^-dependent inhibition of NMDAR currents by desipramine (DES). **(A)** Currents activated by 100 μM NMDA + 10 μM Gly recorded at −70 mV in the presence of 0 or 1 mM Ca^2+^ in the bathing solution in the absence of DES (0) and the presence of rising DES concentrations ([DES]) indicated by numbers at the corresponding level of currents (in μM). Currents recorded in 1 mM Ca^2+^ from BAPTA loaded neurons do not reveal NMDAR desensitization. **(B)** Concentration-inhibition curves for DES of currents activated by NMDA recorded at −70 mV in the presence of different [Ca^2+^] in the bathing solution. Symbols show mean values ± S.E.M of the relative amplitudes of currents (I_b_/I_c_) in the presence (I_b_) and absence (I_c_) of different DES concentrations ([DES]) from 10 to 15 measurements. Solid lines are the fits to the data with the Hill equation (Eq. 1). **(C)** Dependence of IC_50_ for DES inhibition of NMDAR currents on extracellular [Ca^2+^] obtained from experiments illustrated in panel **(B)**. Data from each experiment (symbols) and mean values ± S.E.M. are shown. The IC_50_ values are presented in Table 1. An *e*-fold change in IC_50_ is achieved by a shift of [Ca^2+^] of 0.59 ± 0.07 mM. Increasing extracellular [Ca^2+^] from 0 to 1 mM results in significant (****, *p* < 0.0001, *n* = 10, ANOVA) decrease of IC_50_, while cytosolic BAPTA abolishes this effect.

First, we performed experiments in which the effects of different [Ca^2+^] on the potency of NMDAR inhibition by DES and CLO were tested at a single V_m_. The concentration that cause 50% of the maximal inhibition of currents (IC_50_) of both substances was measured for extracellular [Ca^2+^] of 0, 1 and 4 mM, at V_m_ = −70 mV. Increasing DES concentrations were applied on the top of the steady-state currents induced by 100 μM NMDA (Figure 2A). When reached the steady state in the presence of each DES concentration, amplitudes of currents were measured to plot the concentration-inhibition curve (Figure 2B). The IC_50_ of the inhibition of NMDAR currents was estimated by fitting the concentration-inhibition curves obtained in each experiment with Hill equation (Eq. 1). The mean IC_50_ values are presented in Table 1. The largest IC_50_ value of 8.3 μM was obtained in the calcium-free solution (Figure 2C). With an increase of [Ca^2+^] to 1 mM, the DES IC_50_ decreased of about five-folds, to 1.75 μM. However, the further increase of [Ca^2+^] to 4 mM did not significantly change the IC_50_ value. In accord to ATL data, the Ca^2+^-dependent NMDAR inhibition at a single V_m_ manifests an exponential function (Stepanenko et al., 2019). We, therefore, fitted the DES data by Eq. 6. The sharpness of the dependence estimated as an *e*-fold change of the IC_50_ value was achieved with a [Ca^2+^] shift of 0.59 ± 0.07 mM, which is similar to the sharpness found for ATL (Stepanenko et al., 2019). Thus, the presence of extracellular Ca^2+^ significantly enhances NMDAR inhibition by DES.

**TABLE 1:** The IC_50_ values of the inhibition of NMDAR currents by TCAs.

	V_m_	IC_50_ (µM, [Ca^2+^] = 0)	IC_50_ (µM, [Ca^2+^] = 1 mM)	IC_50_ (µM, [Ca^2+^] = 4 mM)
DES	−100 mV	5.06 + 0.71 (*n* = 11)	0.89 + 0.11 (*n* = 12) ****	
	−70 mV	8.30 + 1.27 (*n* = 10)	1.75 + 0.16 (*n* = 15) ****	1.81 + 0.16 (*n* = 11) ****
	−30 mV	27.0 + 1.9 (*n* = 11)	4.85 + 0.37 (*n* = 15) ****	
		−70 mV (+BAPTA)		11.1 + 1.2 (*n* = 11) *ns*	
	CLO	−100 mV	9.88 ± 1.22 (*n* = 5)	11.8 ± 2.4 (*n* = 13)	
		−70 mV	23.8 ± 4.4 (*n* = 17)	20.6 ± 1.8 (*n* = 13)	7.1 ± 1.4 (*n* = 8) ^§§§§^
		−30 mV	47.3 ± 5.6 (*n* = 12)	44.2 ± 7.1 (*n* = 11)	

****- the data are significantly different from values obtained in [Ca^2+^] = 0 at V_m_ = −70 mV (*p* < 0.0001, 1 way ANOVA), and at V_m_s of −30 mV and −100 mV (Student’s two-tailed *t*-test). ^§§§§^ - the value is significantly different from data obtained in 0 and 1 mM [Ca^2+^] at V_m_ = −70 mV (*p* < 0.0001, 1 way ANOVA with Bonferroni multiple comparisons test). *ns* – the value does not differ significantly from those obtained at [Ca^2+^] = 0, V_m_ = −70 mV (Student’s two-tailed *t*-test, *p* > 0.05).

This observation raises a question whether Ca^2+^ acts from the outside or Ca^2+^ entering through open channels of activated NMDARs contributes to DES effects acting from the inside of neurons, which may promote, for instance, the calmodulin dependent desensitization of NMDARs (for review Sibarov and Antonov 2018). To clarify this issue, measurements of the DES IC_50_ in the presence of 1 mM [Ca^2+^] on neurons loaded with cytosolic Ca^2+^ chelator BAPTA were undertaken. The currents activated by 100 μM NMDA under these particular conditions did not exhibit desensitization as well as in the absence of extracellular Ca^2+^ ([Ca^2+^] = 0, Figure 2A). On BAPTA-loaded neurons, the IC_50_ value of DES increased from 1.75 to 11.1 μM (Table 1) and became similar to the IC_50_ value of 8.3 μM obtained in the absence of extracellular Ca^2^ (Figures 2B,C). From these experiments, we can conclude that the IC_50_ of DES depends on the level of free Ca^2+^ in the cytosol and the chelation of cytosolic Ca^2+^ abolishes the Ca^2+^-dependent NMDAR inhibition.

**FIGURE 3:**
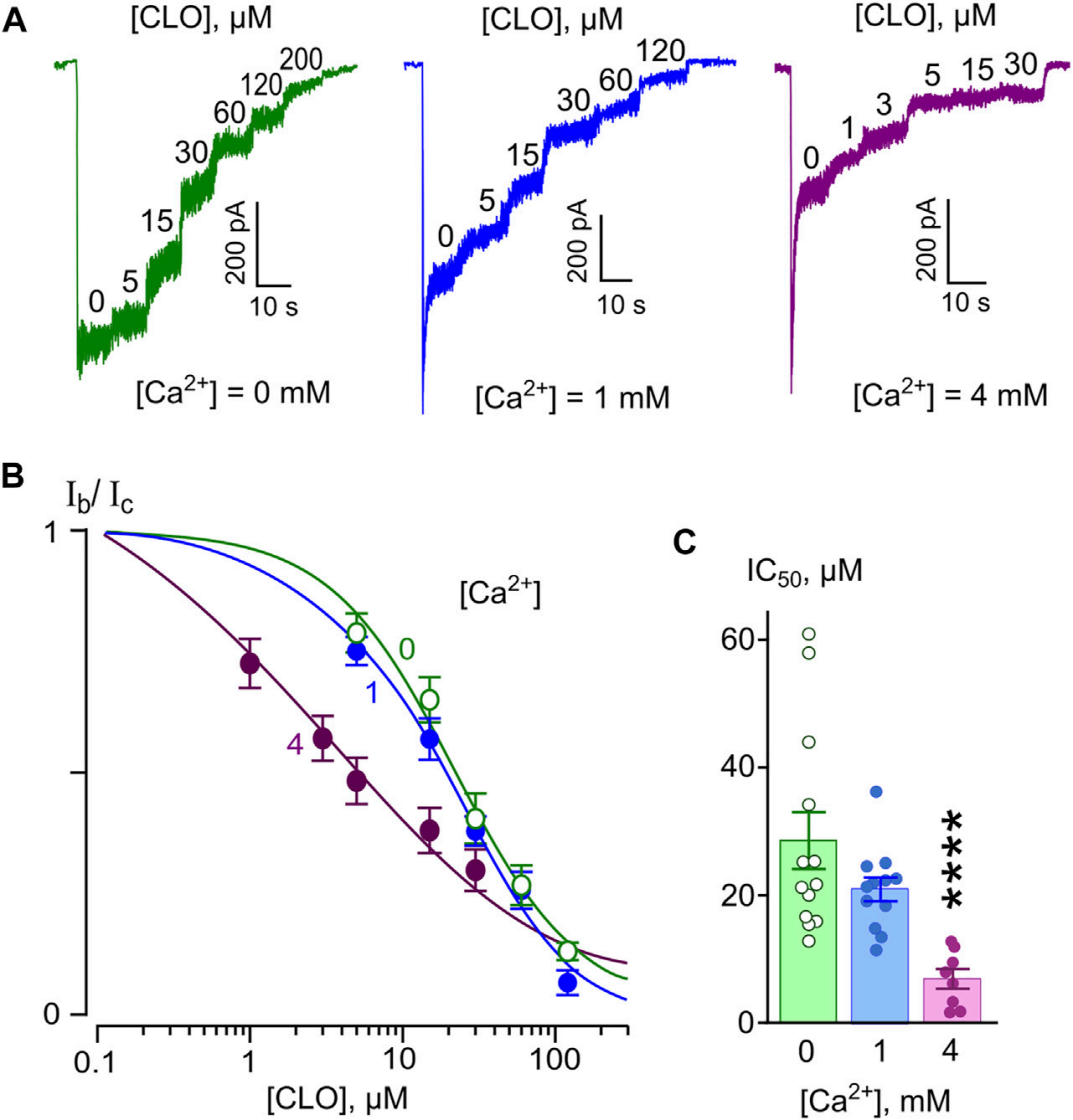
Ca^2+^-dependent inhibition of NMDAR currents by clomipramine (CLO). **(A)** Currents activated by 100 μM NMDA + 10 μM Gly recorded at −70 mV in the presence of 0, 1 or 4 mM Ca^2+^ in the bathing solution in the absence of CLO (0) and the presence of rising CLO concentrations ([CLO]) indicated by numbers at the corresponding level of currents (in μM). **(B)** Concentration-inhibition curves for CLO for currents activated by NMDA recorded at −70 mV in the presence of different [Ca^2+^] in the bathing solution. Symbols show mean values ± S.E.M of the relative amplitudes of currents (I_b_/I_c_) in the presence (I_b_) and absence (I_c_) of different CLO concentrations ([CLO]) from 5 to 17 measurements. Solid lines are the fits to the data with the Hill equation (Eq. 1). **(C)** Dependence of IC_50_ for CLO inhibition of NMDAR currents on extracellular [Ca^2+^] obtained from experiments illustrated in panel **(B)**. Data from each experiment (symbols) and mean values ± S.E.M. are shown. The IC_50_ values are presented in Table 1. An *e*-fold change in IC_50_ is achieved by a shift of [Ca^2+^] of 5.5 ± 1.8 mM. Increasing extracellular [Ca^2+^] from 0 to 1 mM does not produce decrease of IC_50_, but further increase to 4 mM [Ca^2+^] significantly decreases IC_50_ (****, *p* < 0.0001, *n* = 8, ANOVA).

In similar series of experiments, effects of different CLO concentrations were tested on currents activated by NMDA (Figure 3A). The concentration-inhibition curves were plotted for currents recorded in the presence of 0, 1, and 4 mM [Ca^2+^] (Figure 3B). The IC_50_s obtained from these experiments are summarized in Table 1. The IC_50_ values for CLO obtained at [Ca^2+^] = 0 and [Ca^2+^] = 1 mM were 23.8 and 20 µM (Table 1), correspondently, and did not differ significantly. An increase of [Ca^2+^] to 4 mM caused a significant decrease in the IC_50_ value of CLO to 9.88 µM (Table 1; Figure 3C). Fitting the data with Eq. 6 yielded an *e*-fold change of the IC_50_ value achieved with a [Ca^2+^] shift of 5.5 ± 1.8 mM. Therefore CLO manifests an order of magnitude lesser sharp Ca^2+^-dependence of the IC_50_ of NMDAR inhibition than DES.

From these experiments, it became clear that the pharmacological action of DES and CLO does not fit the traditional open-channel block model because of the Ca^2+^-dependent process. In the next series of experiments, we analyzed if DES and CLO still show the dependence of the IC_50_ of NMDAR block on membrane voltage, which is an important feature of the open-channel block.

### The Kinetics of NMDAR Open-Channel Trapping Block by Desipramine

**FIGURE 4:**
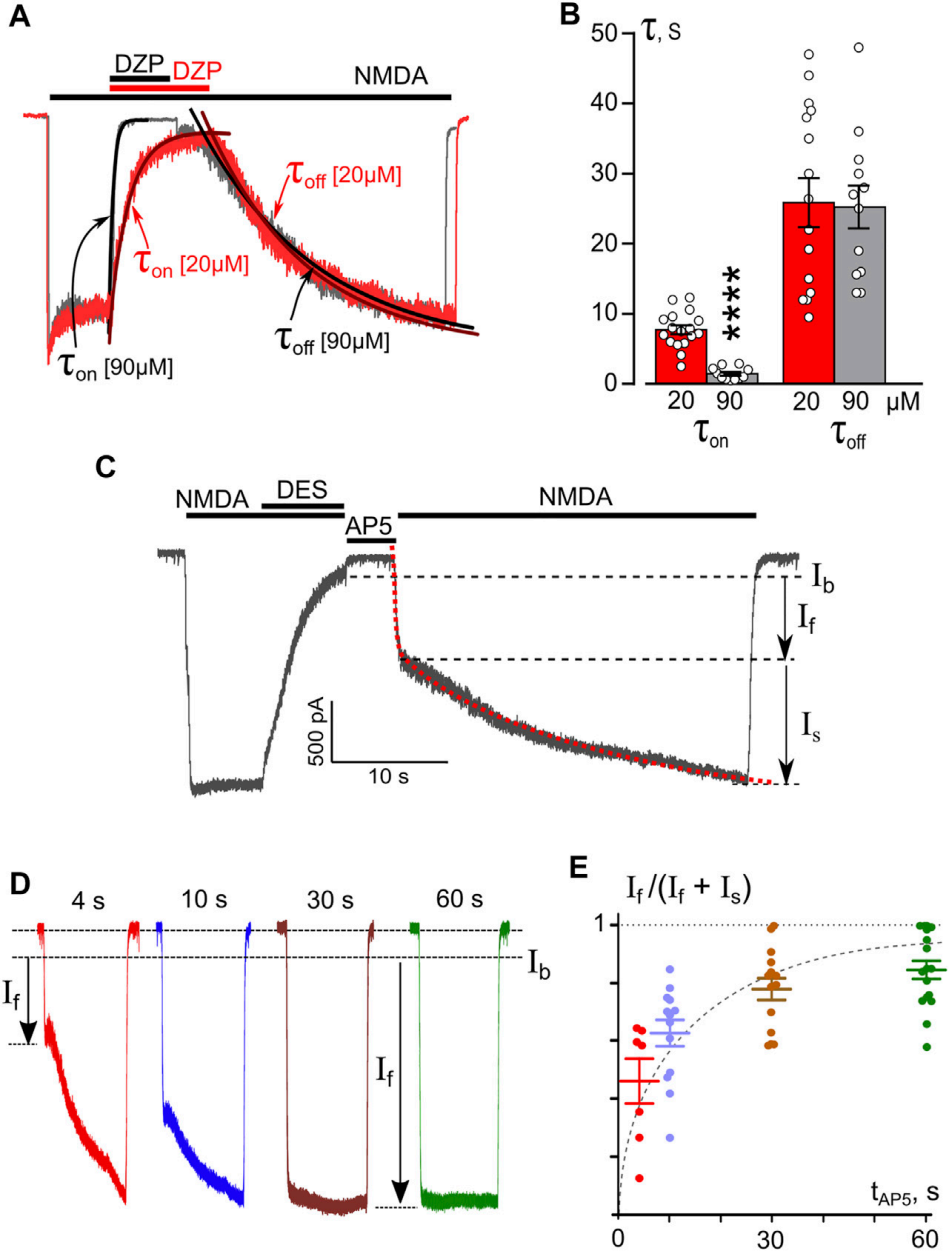
Temporal characteristics of open-channel and trapping block of NMDARs by DES. **(A)** An overlay of normalized currents activated by 100 μM NMDA + 10 μM Gly recorded at −70 mV in the nominal absence of Ca^2+^ ([Ca^2+^] = 0). 20 μM or 90 μM DES was applied at a steady state. Applications of agonist and drug are shown above the traces as bars. The onset and offset of DES block of currents were well fitted by a single exponential function (smooth lines through corresponding currents), yielding time constants for block (τ_on_) and unblock (τ_off_). **(B)** Histogram representing a comparison of time constants for block and unblock obtained for 20 and 90 μM DES at −70 mV. Values (circles) from each experiment, illustrated in panel **(A)**, and mean values ± S.E.M. are compared. **** Data are significantly different (*p* < 0.0001, Student’s two-tailed *t*-test). The K_d_ value for DES, calculated using Eqs 3–5, is 4.7 ± 1.9 μM. **(C)** An example of whole-cell currents activated by NMDA recorded in a neuron at −70 mV in nominal absence of Ca^2+^. When the current reached a steady state, 90 μM DES was added for 7 s, which caused a decrease of the current. After a washout of agonists and DES of duration (tAP5) 4, 10, 30 or 60 s with a bathing solution containing 50 μM AP5, a testing application of NMDA was given. The rise time of the current activated by the testing application was well fitted by a double exponential function (Eq. 7, red dotted line) that yielded amplitudes of fast (I_f_) and slow (I_s_) components and their time constants. (τ_f_ and τ_s_). **(D)** Currents from a neuron activated by a testing application recorded at −70 mV in the nominal absence of Ca^2+^ after washout durations of 4, 10, 30, and 60 s. Arrows indicate amplitudes of fast component (I_f_) of current responses. **(E)** The dependence of I_f_ contribution on the amplitude of testing current measured as I_f_/(I_f_ + I_s_) on a delay duration (t_AP5_). Circles are single experimental measurements, from which mean values ± S.E.M. were calculated. Single exponential fit for mean values yields the time constant of 7.7 s for DES molecule to escape the trapping block in NMDAR.

To study the kinetics of open-channel block of NMDARs by DES, we performed experiments at a single V_m_ = −70 mV using a Ca^2+^-free bathing solution to avoid the influence of the Ca^2+^-dependent process. DES was applied at a steady state of NMDAR currents at high (90 μM) or low (20 μM) concentrations (Figure 4A). DES-induced block corresponds well to basic predictions of an open-channel block. The block onset (τ_on_) and offset (τ_off_) were well fit by a single exponential function (Eq. 2). The τ_on_ value decreased with a concentration increase, whereas the τ_off_ value did not depend on DES concentration (Figure 4B). The rate constants of block and unblock calculated on the basis of Eqs 3, 4 were k^+^ = 0.008 ± 0.002 s^−1^ μM^−1^; k^−^ = 0.039 ± 0.005 s^−1^, correspondently. Accordingly, the equilibrium dissociation constant of DES K_d_ = 4.7 ± 1.9 μM (*n* = 15, Eq. 5), which is not far from the IC_50_ value of 8.3 μM obtained in a Ca^2+^-free solution (Figure 2).

The absence in records of “tail currents” upon the simultaneous removal of agonists and a blocker may speak in favor of trapping of the blocking molecule inside of the pore (Costa and Albuquerque 1994; Vorobjev and Sharonova 1994; Benveniste and Mayer 1995; Sobolevsky et al., 1999). We, therefore, further tested the possibility of trapping of DES in the pore of NMDARs by the channel closure. A series of experiments were carried out following the protocol used previously for ATL (Stepanenko et al., 2019). After reaching an equilibrium of NMDA-mediated currents, 90 μM DES was applied to the cell for 10 s until the development of a stable block with a small amount of residual current (I_b_) (Figure 4C). Then, the solution with the agonist and DES was washed off, and to avoid accidental opening of NMDARs, 50 μM AP5, a competitive antagonist of the NMDAR glutamate binding site, was applied. After finishing the AP5 application, the NMDA test solution was again applied to the cells. This sequence of applications was performed several times per cell with varying durations of AP5 delivery - 4, 10, 30, 60 s (Figure 4C). The biphasic current response to the NMDA test application was composed of fast and slow components. Fitting the current with a double-exponential function (Eq. 7, Figure 4C dotted line) yielded the rise time rates and amplitudes (I_f_ and I_s_) for the fast and slow components. The time constants did not depend on the washout duration. The fast component rate describes the opening of unblocked channels in response to agonist action and has an I_f_ amplitude (Figure 4D). The time constant of the slow component (τ_off_) is 25 s and corresponds to the kinetics of DES release by blocked channels. An increase of I_f_ in the total current amplitude (I_f_ + I_s_) with the channel closed state time may indicate a gradual release of blocker molecules from the channels in the closed state (Figure 4E). Single exponential approximation of the data at Figure 4E (dashed line) elicits the time constant of 7.7 ± 1.8 s for DES escape from trapping, representing the quantitative characteristic of DES partial trapping block.

### Voltage-dependent Open-Channel Block of NMDARs by TCAs

**FIGURE 5:**
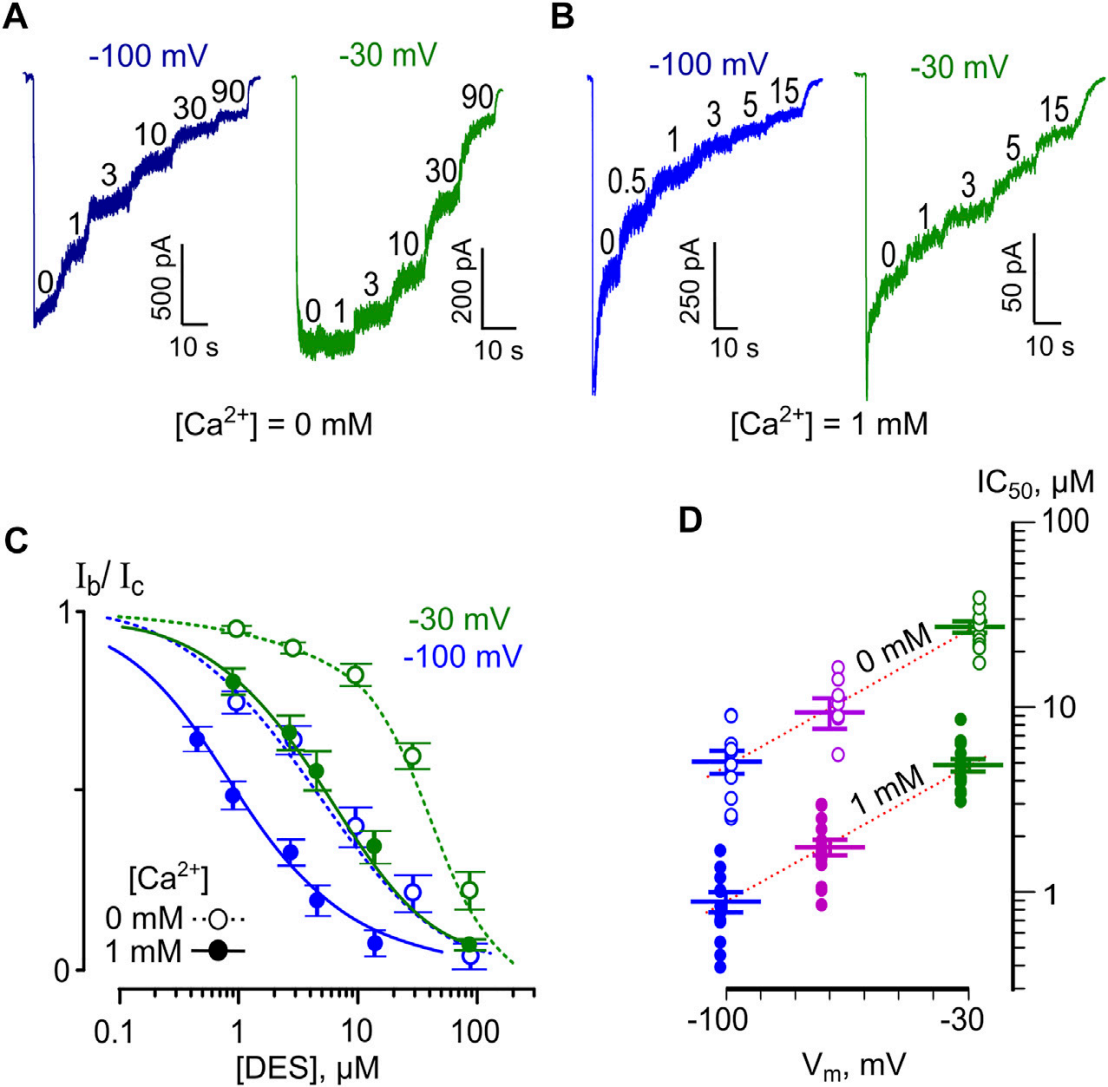
Voltage dependence of open channel block of NMDARs by DES. **(A)** Currents activated by 100 μM NMDA + 10 μM Gly recorded in 0 mM Ca^2+^ at −100 mV and −30 mV in the absence of DES (0) and the presence of rising DES concentrations ([DES]) indicated by numbers at the corresponding level of current (in μM). **(B)** Currents activated by NMDA recorded in 1 mM Ca^2+^ at −100 mV and −30 mV in the absence of DES (0) and in the presence of rising DES concentrations ([DES]) indicated by numbers at the corresponding level of current (in μM). **(C)** Concentration-inhibition curves for DES of currents activated by NMDA recorded at −100 mV and −30 mV in the presence of 0 or 1 mM Ca^2+^. Symbols (empty circles for 0 mM Ca^2+^ and solid circles for 1 mM Ca^2+^) depict mean values ± S.E.M of relative amplitudes of currents (I_b_/I_c_) measured in the presence (I_b_) and absence (I_c_) of different DES concentrations from 10 to 15 experiments. Lines are the fits to the data with the Hill equation (Eq. 1) – dotted lines for 0 mM Ca^2+^ and solid lines for 1 mM Ca^2+^. **(D)** Voltage dependence of the IC_50_ value for DES inhibition of NMDA-activated currents. Values (empty circles for 0 mM Ca^2+^ and solid circles for 1 mM Ca^2+^) from each experiment and mean values ± S.E.M. are shown. The dotted line depicts fit to the data with Eq. 6, which yielded IC_50_(0) = 60 ± 5.7 µM and an e-fold change of the IC_50_ of 37 ± 3.4 mV.

To evaluate the voltage-dependence of the open-channel NMDAR block, we measured the IC_50_ of DES at three V_m_s of −100, −70, and −30 mV. Since increasing extracellular [Ca^2+^] from 0 to 1 mM caused a 5-fold drop of DES IC_50_, the voltage-dependence was studied at both [Ca^2+^]. Examples of currents recorded at outermost V_m_s in Ca^2+^-free solution and in [Ca^2+^] = 1 mM are shown in Figures 5A,B, respectively. The increase of DES concentration induced strengthening of the inhibition of NMDA-activated currents. In addition, similar DES concentrations evoked a lesser decrease of NMDAR currents at depolarized V_m_s. The concentration-inhibition curves (Figure 5C) reveal the voltage-dependence of the block since the DES IC_50_ value decreased substantially with hyperpolarization, and the Ca^2+^-dependence of block, since in the presence of 1 mM [Ca^2+^] the block was more pronounced. The dependence of block on V_m_ is illustrated in Figure 5D. A shift in V_m_ from −30 to −100 mV leads to a 5.4-fold IC_50_ decrease regardless of extracellular calcium (Figure 5D; Table 1). While the proper characteristic of the NMDAR open-channel block could be obtained in the absence of Ca^2+^, the Ca^2+^-dependent inhibition is V_m_ resistant and enhances the apparent NMDAR inhibition. This suggests that the V_m_- and Ca^2+^-dependent effects have different origins and occur independently.

Interpretation of the voltage-dependence of the DES open-channel block within the framework on the Woodhull model (Eq. 6) yields an *e*-fold change of the IC_50_ value that could be achieved with a 37 mV shift of V_m_. Fitting the apparent data shown in Table 1 with Eq. 6 yields IC_50_(0 mV) = 60 ± 5.7 µM (*n* = 10) and an *e*-fold change of 36.9 ± 3.4 mV for the IC_50_ voltage-dependence and an *e*-fold change of 0.59 ± 0.07 mM for the IC_50_ Ca^2+^ dependence.

**FIGURE 6:**
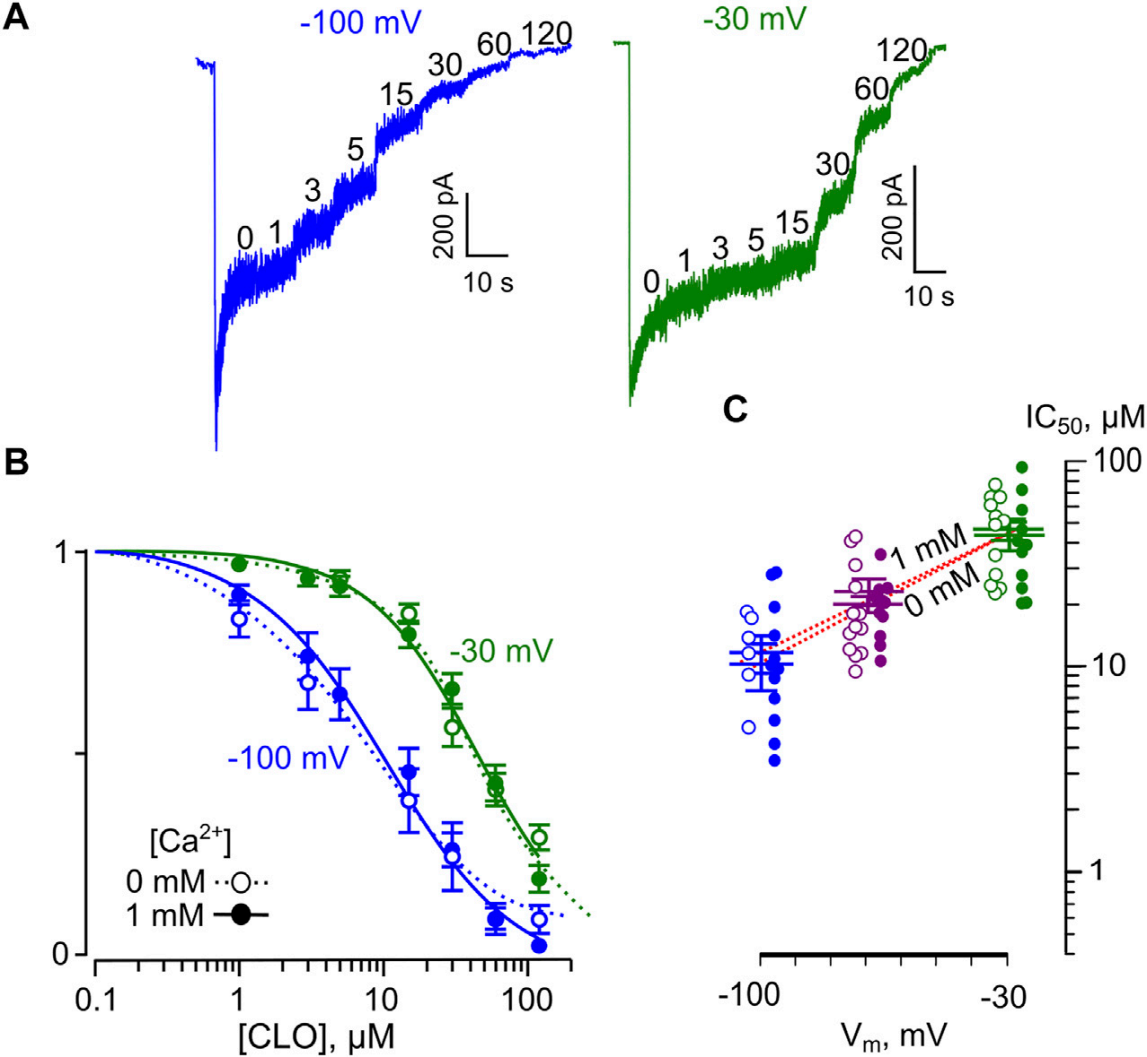
Voltage dependence of open channel block of NMDARs by CLO. **(A)** Sample currents activated by 100 μM NMDA + 10 μM Gly recorded in 1 mM Ca^2+^ at −100 mV, and −30 mV in the absence of CLO (0) and the presence of rising CLO concentrations ([CLO]) indicated by numbers at the corresponding level of current (in μM). **(B)** Concentration-inhibition curves for CLO for currents activated by NMDA recorded at −100 mV, and −30 mV in the presence of 0 or 1 mM Ca^2+^. Symbols (empty circles for 0 mM Ca^2+^ and solid circles for 1 mM Ca^2+^) depict mean values ± S.E.M of relative amplitudes of currents (I_b_/I_c_) measured in the presence (I_b_) and absence (I_c_) of different CLO concentrations from 5 to 17 experiments. Lines are the fits to the data with the Hill equation (Eq. 1). **(C)** Voltage dependence of the IC_50_ value for CLO inhibition of NMDA-activated currents. Values (empty circles for 0 mM Ca^2+^ and solid circles for 1 mM Ca^2+^) from each experiment and mean values ± S.E.M. are shown. The dotted line depicts fit to the data with Eq. 6, which yielded IC_50_(0) = 91 ± 8.8 µM and an *e*-fold change of the IC_50_ of 50 ± 5.5 mV.

We studied the voltage-dependence of the NMDAR block by CLO using similar approaches as for DES. Examples of currents obtained at V_m_s of −30 and −100 mV in [Ca^2+^] = 1 mM are illustrated in Figure 6A. The averaged concentration-inhibition curves for −30, and −100 mV were similar for both 0 and 1 mM external [Ca^2+^] (Figure 6B), which in addition could become obvious from the analysis of the raw data (Table 1). The V_m_-dependence for CLO IC_50_ for all conditions under study is shown in Figure 6C. The voltage dependence of the CLO IC_50_ values obtained by fitting the raw data with Eq. 6 yields IC_50_ (0 mV) = 91 ± 8.8 µM (*n* = 5) and an *e*-fold change of 50 ± 5.5 mV regardless of the presence of extracellular Ca^2+^.

These unmask a great difference in the pharmacological action on NMDARs between DES and CLO. First, the voltage-dependence of the DES open-channel block of NMDARs is much stronger than those for CLO and, second, unlike DES, CLO does not demonstrate the Ca^2+^-dependent inhibition within the range of physiologically relevant [Ca^2+^].

## Discussion

### Ca^2+^-dependent Effects on NMDARs

The Ca^2+^-dependent inhibition of NMDAR currents described here is much more pronounced for DES than for CLO (Table 2). This observation suggests that the origin of the Ca^2+^-dependence of these two compounds may differ.

**TABLE 2:** Parameters of TCA voltage- and calcium-dependences of “comorbid” blockade of NMDARs.

	IC_50_(0 mV)	e-fold change [Ca^2+^]	e-fold change V_m_
ATL^a^	321 µM	0.63 mM	54 mV
DES	60 ± 5.7 µM	0.59 ± 0.07 mM	36.9 ± 3.4 mV
CLO	91 ± 8.8 µM	5.5 ± 1.8 mM	50 ± 5.5 mV

a Data are recruited from (Stepanenko et al., 2019).

One possible mechanism could represent the enhancement of the Ca^2+^-dependent desensitization (CDD) of NMDARs which earlier was assumed to explain the Ca^2+^-dependence of ATL effects on NMDARs (Stepanenko et al., 2019). CDD results from binding Ca^2+^ ions entering through the activated NMDAR channels with calmodulin and interaction of the latter with the intracellular domain of NMDARs (Ehlers et al., 1996; Zhang et al., 1998; for review Sibarov and Antonov 2018). CDD manifests in a decrease of macroscopic currents, despite the continued presence of the agonists (Zhang et al., 1998; Sessoms-Sikes et al., 2005). The removal of Ca^2+^ from the extracellular solution abolished NMDAR CDD suggesting that Ca^2+^ entered neurons from the outside is an indispensable condition for CDD development. Preventing Ca^2+^ binding to calmodulin by chelation of free intracellular Ca^2+^ with BAPTA can abolish CDD for both GluN1/GluN2A, and GluN1/GluN2B receptor subtypes (Iacobucci and Popescu, 2020). In our experiments, loading of BAPTA into the cytosol increased DES IC_50_ to the values observed in a calcium-free extracellular solution. Similar dependence on intracellular [Ca^2+^] was reported previously for ATL (Stepanenko et al., 2019). Thus, the Ca^2+^-dependent effect on NMDARs of DES and ATL conforms to the intracellular [Ca^2+^] and manifests itself only when the CDD process is not blocked by the lack of extracellular or intracellular free Ca^2+^.

The inhibition of NMDARs by DES has been widely studied previously (Reynolds and Miller 1988; Sernagor et al., 1989; Nothdurfter et al., 2013; Barygin et al., 2017). The DES IC_50_ values varying from 1.4 to 9.8 μM were reported. The great variability of the value is likely could be determined by experimental conditions in which a different contribution of open-channel block and Ca^2+^-dependent inhibition is expected. For example, to activate NMDAR currents in some experiments (Sernagor et al., 1989), 5 μM NMDA was used, which is much smaller than the EC_50_ of NMDAR activation. Since the Ca^2+^ intracellular accumulation and CDD depend on the NMDAR open probability, the usage of low agonist concentration may not allow to cause CDD. As a result, the open-channel block of NMDARs represents the only mechanism of DES action. Indeed the DES IC_50_ of 9.8 μM and an *e*-fold of 36 mV were found in this study despite the physiological level of extracellular Ca^2+^. This coincides well with our data (IC_50 =_ 8.3 μM, *e*-fold 37 mV) obtained in a calcium-free solution in the absence of CDD. In agreement, 7.41 μM DES displaces MK-801 from the NMDAR channels due to the competition of these compounds for the ion pore (Reynolds and Miller 1988).

When studied in the presence of extracellular Ca^2+^ and receptor saturating concentration of NMDA (100 μM), the DES IC_50_ at V_m_ = −70 mV was found to be 2.7 µM (Nothdurfter et al., 2013) and 1.4 µM (Barygin et al., 2017), which is compatible with the values of about 1.7 µM obtained in our experiments. If ATL and DES may affect the efficacy of Ca^2+^ export from neurons by Na^+^/Ca^2+^-exchanger increasing the pre-membrane intracellular free Ca^2+^ concentration, then an enhancement of CDD may contribute to the Ca^2+^-dependent and V_m_-resistant inhibition of NMDARs. Interestingly memantine, a widely used NMDAR channel blocker, also inhibits Na^+^/Ca^2+^-exchanger (Brittain et al., 2012), which probably contributes to memantine-induced stabilization of NMDAR in CDD state (Glasgow et al., 2017). The Ca^2+^ dependence of NMDAR inhibition by memantine (Glasgow et al., 2017), ATL (Stepanenko et al., 2019), and DES was abolished by cytosolic BAPTA. Therefore modulation of Ca^2+^ interaction with intracellular receptor domain may explain the voltage-resistant Ca^2+^-dependent effects of these substances on NMDARs.

Beyond the CDD, which is similar for GluN2A- and GluN2B-containing receptors (Iacobucci and Popescu, 2020), the direct voltage-independent effects of extracellular Ca^2+^ on recombinant GluN1/GluN2A receptor conductance and gating are also demonstrated (Watanabe et al., 2002; Maki and Popescu 2014). Even in NMDARs without the GluN1 intracellular domains, Ca^2+^ ions inhibit NMDAR ion fluxes through direct interactions with extracellular receptor residues located in the external vestibule of NMDARs with the IC_50_ of 2.4 mM (Maki and Popescu 2014). The direct effects of extracellular Ca^2+^ on NMDARs are independent on intracellular Ca^2+^ sensitive domains of GluN1 (Maki and Popescu 2014). Perhaps this mechanism could be responsible for the Ca^2+^-dependence of CLO NMDAR inhibition which is rather weak since an *e*-fold change of the CLO IC_50_ could be achieved with a 5.5 mM shift of [Ca^2+^].

### Structure-Activity Relationships of Ca^2+^-dependent Inhibition

Whereas the molecular structures of TCAs studied here are rather similar, DES and CLO drastically differ by the sharpness of Ca^2+^-dependent NMDAR inhibition characterized by an *e*-fold change value that was almost an order of magnitude smaller for DES than for CLO (Table 2). The consistence of the DES and ATL effects allows us to conclude that the difference in chemical structures of DES and ATL do not matter for the effect and both compounds match to produce the Ca^2+^-dependent NMDAR inhibition. On the contrary, a particle of the CLO molecule not shared with both DES and ATL is responsible for the loss of this effect (Table 3). This is a chlorine atom. Presumably, the presence of the chlorine atom makes the folded conformation of molecules highly probable when the aliphatic side-chain moves toward the chlorine substituted phenyl ring due to electrostatic attraction between the positively charged amino group and the chlorine atom in the CLO molecule, which prevents inherent extended side-chain conformations of other TCAs (Heimstad et al., 1991). Looking ahead, it should be noted that this property of the CLO molecules does not matter for the open-channel block. Anyway, a direct study of the molecular target involved in the Ca^2+^-dependent NMDAR inhibition could solve this riddle.

**TABLE 3:** Structure-activity relationships of NMDAR inhibition by TCAs.

		Voltage-dependence	Potency at 0 mV	Ca^2+^-dependence
R_1_	-NH(CH_3_)_2_	−	no effect	no effect
	-NH_2_CH_3_	+		
R_2_	C	no effect	−	no effect
	N		+	
R_3_	Cl	no effect	no effect	−
	no Cl			+

R_1,_ R_2_, R_3_ are marked in Figure 1. R_1_ and R_2_ affect the open-channel block, while R_3_ is in charge of the Ca^2+^-dependent inhibition. “−,” “+” show a modality of the effects (reduction or enhancement).

### Structure-Activity Relationships of NMDAR Open-Channel Block by TCAs

To come out with the “proper” parameters of NMDAR open-channel block by DES and CLO and to avoid a concomitant Ca^2+^-dependent inhibition under the physiological conditions ([Ca^2+^] = 1 or 2 mM, V_m_ = −70 mV), we first studied this mechanism at [Ca^2+^] = 0 and V_m_ = −70 mV, using a similar approach as for ATL (Stepanenko et al., 2019). Then the dependence of the parameters on V_m_ and [Ca^2+^] was tested since the V_m_-dependence represents an inherent property of the block (Table 2).

TCA potencies as open-channel blockers can be juxtaposed based on IC_50_ (0 mV) values, derived from measurements of the V_m_-dependence. Clearly, while DES (with the value of 60 µM) and CLO (of 90 µM) are rather similar, these compounds are almost 5-folds more powerful than ATL. This difference could be determined by a low rate of DES dissociation (τ_off_ is about 25 s) from channels which is of about 5-folds lesser than the rate for ATL, suggesting that the molecule of ATL occupies the channel at the least one-fifth of the occupancy time by DES and presumably CLO during the open-channels block. DES, as well as ATL (Stepanenko et al., 2019), demonstrates partial trapping open-channel block that makes them similar to amantadine and memantine (Blanpied et al., 1997), suggesting that the blocking molecule can be trapped inside of the pore by the gate closure and can escape the channel in the closed state at some certain rate. The time constant of the escape for DES was about 7.7 s compared to about 1.2 s reported to ALT (Stepanenko et al., 2019). The slower escape of DES from the closed channel than of ATL is also connected with some peculiarity of its chemical structure. DES and CLO, therefore, demonstrate the high-affinity open-channel block of NMDARs, which most likely is determined by slow rates of dissociation both from open and closed states of channels. Definitely, this should somehow be related to a shared common element of the chemical structure of these TCAs, which differs them from ATL. This is a tricyclic radical, which in the case of DES and CLO, contains the nitrogen atom in contrast to the carbon atom of ATL (Table 3). The substitution of nitrogen for a carbon atom in the central aromatic ring eliminates a double bond in the aliphatic chain, which elevates the probability of extended side-chain conformation as well as the flexibility of the TCA molecule (Heimstad et al., 1991). Presumably, these underlay a better geometric arrangement of TCAs and their accommodation within the binding site in the NMDAR ion pore.

The traditional interpretation of open-channel block within the framework of the Woodhull model implies that the voltage dependence is determined by the charge of blocking molecules and a deepness of the binding site location in the channel on which the electric field drop occurs. For example, Mg^2+^ demonstrates a highly voltage-dependent block of NMDARs (Nowak et al., 1984). The Mg^2+^ binding site, however, is located close to the middle of the NMDAR channel (Antonov and Johnson 1999). As for molecules, singly charged IEM-compounds (Antonov et al., 1995) exhibit different voltage-dependence of NMDAR open-channel block that regards to a steric size of the side-amino group; as the size is smaller, the molecule can get deeper into the channel (Antonov and Johnson 1996; Antonov et al., 1998). In accord to the structure-activity relationship for the efficacy of open-channel block described above, ATL and CLO, which have the same aliphatic side-chain amino group (-NH(CH_3_)_2_), reveal weaker voltage-dependence than those for DES containing a smaller amino group (-NH_2_CH_3_) (Table 3). This observation is consistent with data on IEM-compounds (Antonov and Johnson 1996; Antonov et al., 1998) and allows us to assume that the smaller size amino group may permeate deeper in the channel. Accordingly, an *e*-fold change of DES IC_50_ requires ∼1.5 folds smaller shift in V_m_ than those for ATL or CLO (Table 2). The δ value characterizing the fraction of the membrane electric field that the molecule traverses to reach the binding site inside the NMDAR channel places the DES binding site to 0.71 from the outside, which is greater than δ = 0.51 for ATL reported previously (Stepanenko et al., 2019), and CLO studied here. Therefore, considering the TCA binding in the channel with the aliphatic side-chain amino group pointing forward (Stepanenko et al., 2019), the smaller size -NH_2_CH_3_ group of DES, as compared to –NH(CH_3_)_2_ of ATL and CLO, probably facilitates deeper DES penetration to the channel.

### “Comorbid” Blockade of NMDARs by TCAs

**FIGURE 7:**
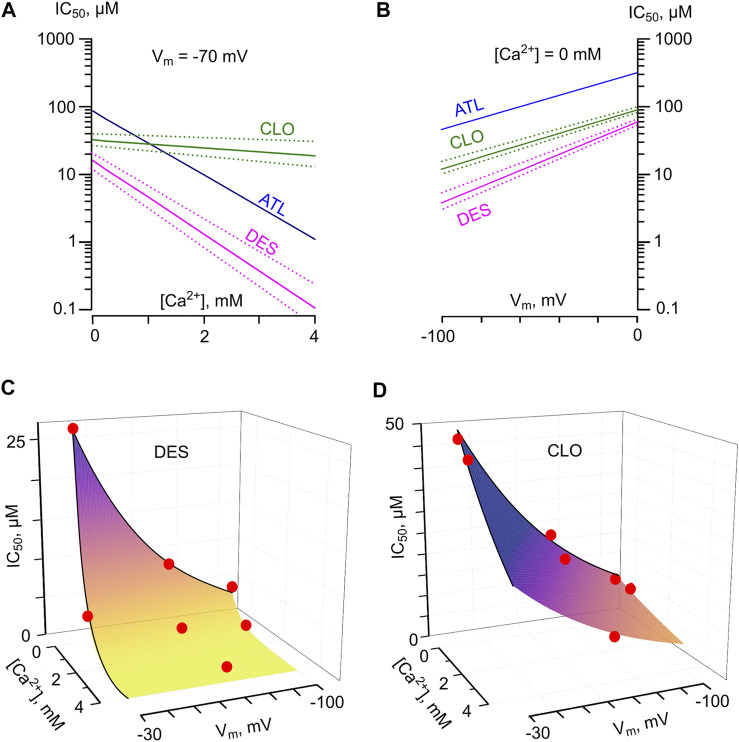
Predicted IC_50_ values for NMDAR inhibition by tricyclic antidepressants as a function of membrane voltage (V_m_) and external [Ca^2+^]. **(A)** The dependence of IC_50_ values for desipramine (DES) and clomipramine (CLO) on [Ca^2+^] derived by fitting the data with Eq. 6 at V_m_ = −70 mV. Dotted lines represent SEMs. The dependence for amitryptiline (ATL) taken from [14] is shown for a comparison. **(B)** The dependence of IC_50_ values for desipramine (DES) and clomipramine (CLO) on V_m_ derived by fitting the data with Eq. 6 obtained in the absence of Ca^2+^. Dotted lines represent SEMs. The dependence for amitryptiline (ATL) taken from (Stepanenko et al., 2019) is shown for a comparison. **(C**,**D)** Predicted values of NMDAR “comorbid” blockade for DES and CLO, correspondently. The IC_50_s were calculated as IC_50_(V_m_, [Ca^2+^]) = IC_50_(0 mV) · exp (V_m_/Y) ·exp(-[Ca^2+^]/X), where X is equal to the [Ca^2+^] value required for an *e*-fold change of IC_50_, and Y is equal to the V_m_ value required for an *e*-fold change of IC_50_. Red circles represent the mean IC_50_ values obtained in experiments. The values used to plot the surfaces are presented in Table 2. The color transition of the surfaces to the yellow exhibits an approach to the concentration range of drugs found during therapy in the blood.

Clearly, the parameters of NMDAR inhibition obtained in experiments result from a combination of two or even more processes. Different structure-activity relationships of TCAs with respect to the Ca^2+^-dependent inhibition and the V_m_-dependent open-channel block of NMDARs suggest that these processes are independent and most likely may represent an action on different molecular targets. The observation that the absence or presence of Ca^2+^-dependent inhibition did not alter the voltage-dependence for DES, CLO as well as ATL (Stepanenko et al., 2019) further supports this assumption. Previously for ATL, we proposed the empirical model (Stepanenko et al., 2019) for a dependence of inhibition on V_m_ and [Ca^2+^] that implies the absence of competition between Ca^2+^- and voltage-dependent effects. The model is described by Eq. 6 IC_50_ (V_m_, [Ca^2+^]) = IC_50_ (0 mV) • exp (V_m_/(*e*-fold change V_m_)) • exp (- [Ca^2+^]/(*e*-fold change [Ca^2+^])). The dependences of TCA IC_50_s on [Ca^2+^] (Figure 7A) and V_m_ (Figure 7B) derived from the exponential fits to the data were polled to calculate predictions of the model. At physiological values of [Ca^2+^], it allows an estimation of IC_50_ values that are consistent to the experimental data for DES (Figure 7C), CLO (Figure 7D), and ATL (Stepanenko et al., 2019). Nevertheless for DES, at [Ca^2+^] of 4 mM and V_m_ = −70 mV, the model predicts IC_50_ = 0.01 μM which deviates from experimental data of 1.8 μM. Therefore, we cannot exclude that the direct extracellular Ca^2+^ action on NMDARs (Watanabe et al., 2002; Maki and Popescu 2014) may contribute to the distortion at physiologically excessive extracellular [Ca^2+^]. This contribution perhaps could be evaluated considering the [Ca^2+^] effect on CLO IC_50_.

At physiological [Ca^2+^] of 2.5 mM and V_m_ = −70 mV, the model described by Eq. 6 predicts the IC_50_ = 0.13 μM for DES and IC_50_ = 1.6 μM for ATL. These IC_50_s correspond well to the TCAs therapeutic concentrations of 0.5–1.5 μM (Vandel et al., 1992), which could be achieved during the treatment of neuropathic pain. CLO and DES share similar efficacy of channel block, but CLO differs by a weak Ca^2+^-dependence of NMDAR inhibition. At [Ca^2+^] = 2.5 mM and V_m_ = −70 mV the model provides CLO IC_50_ = 14 μM, which strongly exceeds CLO therapeutic blood concentrations of 0.3–1 μM (Stern et al., 1980; McTavish and Benfield 1990). Therefore CLO clinical effects probably do not involve NMDAR inhibition.

**FIGURE 8:**
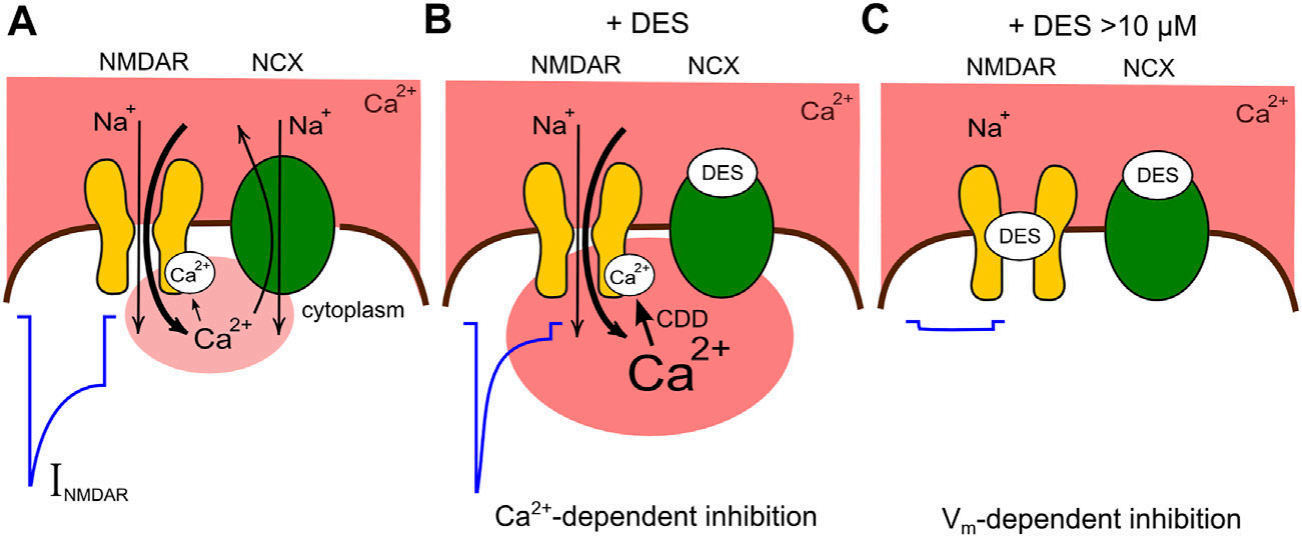
Schematic presentation of the comorbid blockade of NMDARs by DES. **(A)** Functional interaction between NMDARs and Na^+^/Ca^2+^ exchangers (NCX) under control conditions. In the presence of physiologically relevant extracellular [Ca^2+^] the activation of NMDARs is followed by CDD which is proportional to the Ca^2+^ entry *via* ion pores. NCX restricts CDD extruding intracellular Ca^2+^. The current activated by NMDA (I_NMDA_) reveals a moderate CDD. **(B)** DES at therapeutic concentrations inhibits the Ca^2+^ removal by NCX, provokes a local Ca^2+^ accumulation and an enhancement of CDD making the NMDAR current smaller. **(C)** At larger concentration DES causes, in addition, the open-channel block of NMDARs futher suppressing currents activated by NMDA.

Thus, we demonstrated here two components of TCA action on NMDARs, which are represented by the Ca^2+^-dependent inhibition (Figures 8A,B) and the voltage-dependent open-channel block (Figure 8C). We observed that the “pure” channel block of NMDARs by DES, ATL, and CLO occurs at concentrations exceeding the therapeutic ones found in blood plasma (Santos et al., 2017). The contribution of NMDAR channel block to TCA effects against neuropathic pain is probably weaker than those of more potent NMDAR channel blockers with antinociceptive properties like ketamine, memantine (Kurian et al., 2019), and MK-801 (Peterson et al., 2021). However, NMDAR channel blockers demonstrate strong adverse effects limiting their clinical use (Ellison 1995; Kurian et al., 2019). Conversely, the Ca^2+^-dependent inhibition of NMDARs by ATL and DES occurs at therapeutic concentrations and may arise from a disruption of Ca^2+^ export from neurons (Figure 8B) (Lavoie et al., 1990), which in turn modulates CDD of NMDARs (Sibarov et al., 2015; Sibarov et al., 2018). Therefore the enhancement of CDD exaggerates comorbid blockade of NMDARs by TCAs and places their potencies within the range of therapeutic concentrations required for neuropathic pain treatments. Actually, other Na^+^/Ca^2+^-exchanger inhibitors like lithium ions or KB-R7943 demonstrate Ca^2+^-dependent inhibition of NMDARs (Sibarov et al., 2015; Sibarov et al., 2018) and exhibit an antinociceptive effect in neuropathic pain models (Shimizu et al., 2000; Huang et al., 2019). It is likely that Na^+^/Ca^2+^-exchanger and the Ca^2+^-dependent inhibition of NMDARs represent promising targets to treat neuropathic pain pharmacologically.

The contribution of one or other mechanism of NMDAR inhibition is activity-dependent because the voltage-dependent block weakens with glutamate-induced membrane depolarization, but the Ca^2+^-dependent inhibition augments with glutamate elicited Ca^2+^ entry into the cytoplasm of depolarized neurons. These two modes of TCAs action on NMDARs most likely govern the different therapeutic profiles and side effects of these drugs.

## Data Availability

The original contributions presented in the study are included in the article/Supplementary Material, further inquiries can be directed to the corresponding authors.

## References

[B1] Antonov S. M. Gmiro V. E. Johnson J. W. (1998). Binding Sites for Permeant Ions in the Channel of NMDA Receptors and Their Effects on Channel Block. Nat. Neurosci. 1, 451–461. 10.1038/2167 10196542

[B2] Antonov S. M. Johnson J. W. Lukomskaya N. Y. Potapyeva N. N. Gmiro V. E. Magazanik L. G. (1995). Novel Adamantane Derivatives Act as Blockers of Open Ligand-Gated Channels and as Anticonvulsants. Mol. Pharmacol. 47 (3), 558–567. 7535380

[B3] Antonov S. M. Johnson J. W. (1999). Permeant Ion Regulation of N-Methyl-D-Aspartate Receptor Channel Block by Mg(2+). Proc. Natl. Acad. Sci. U S A. 96 (25), 14571–14576. 10.1073/pnas.96.25.14571 10588746 PMC24477

[B4] Antonov S. M. Johnson J. W. (1996). Voltage-dependent Interaction of Open-Channel Blocking Molecules with Gating of NMDA Receptors in Rat Cortical Neurons. J. Physiol. 493 (2), 425–445. 10.1113/jphysiol.1996.sp021394 8782107 PMC1158928

[B5] Barygin O. I. Nagaeva E. I. Tikhonov D. B. Belinskaya D. A. Vanchakova N. P. Shestakova N. N. (2017). Inhibition of the NMDA and AMPA Receptor Channels by Antidepressants and Antipsychotics. Brain Res. 1660, 58–66. 10.1016/j.brainres.2017.01.028 28167075

[B6] Belinskaia D. A. Belinskaia M. A. Barygin O. I. Vanchakova N. P. Shestakova N. N. (2019). Psychotropic Drugs for the Management of Chronic Pain and Itch. Pharmaceuticals (Basel) 12 (2), 99. 10.3390/ph12020099 PMC6631469 31238561

[B7] Benveniste M. Mayer M. L. (1995). Trapping of Glutamate and Glycine During Open Channel Block of Rat Hippocampal Neuron NMDA Receptors by 9-aminoacridine. J. Physiol. 483, 367–384. 10.1113/jphysiol.1995.sp020591 7650609 PMC1157850

[B8] Blanpied T. A. Boeckman F. A. Aizenman E. Johnson J. W. (1997). Trapping Channel Block of NMDA-Activated Responses by Amantadine and Memantine. J. Neurophysiol. 77, 309–323. 10.1152/jn.1997.77.1.309 9120573

[B9] Boikov S. I. Sibarov D. A. Antonov S. M. (2020). Ethanol Inhibition of NMDA Receptors in Calcium-dependent and -Independent Modes. Biochem. Biophys. Res. Commun. 522, 1046–1051. 10.1016/j.bbrc.2019.12.007 31818458

[B10] Brittain M. K. Brustovetsky T. Sheets P. L. Brittain J. M. Khanna R. Cummins T. R. (2012). Delayed Calcium Dysregulation in Neurons Requires Both the NMDA Receptor and the Reverse Na+/Ca2+ Exchanger. Neurobiol. Dis. 46, 109–117. 10.1016/j.nbd.2011.12.051 22249110 PMC3299854

[B11] Bryson H. M. Wilde M. I. (1996). Amitriptyline. A Review of its Pharmacological Properties and Therapeutic Use in Chronic Pain States. Drugs Aging 8, 459–476. 10.2165/00002512-199608060-00008 8736630

[B12] Chen S. R. Zhou H. Y. Byun H. S. Chen H. Pan H. L. (2014). Casein Kinase II Regulates N-Methyl-D-Aspartate Receptor Activity in Spinal Cords and Pain Hypersensitivity Induced by Nerve Injury. J. Pharmacol. Exp. Ther. 350, 301–312. 10.1124/jpet.114.215855 24898266 PMC4109487

[B13] Costa A. C. Albuquerque E. X. (1994). Dynamics of the Actions of Tetrahydro-9-Aminoacridine and 9-aminoacridine on Glutamatergic Currents: Concentration-Jump Studies in Cultured Rat Hippocampal Neurons. J. Pharmacol. Exp. Ther. 268, 503–514. 7507997

[B14] Dharmshaktu P. Tayal V. Kalra B. S. (2012). Efficacy of Antidepressants as Analgesics: A Review. J. Clin. Pharmacol. 52, 6–17. 10.1177/0091270010394852 21415285

[B15] Ehlers M. D. Zhang S. Bernhadt J. P. Huganir R. L. (1996). Inactivation of NMDA Receptors by Direct Interaction of Calmodulin with the NR1 Subunit. Cell 84 (5), 745–755. 10.1016/S0092-8674(00)81052-1 8625412

[B16] Eisenach J. C. Gebhart G. F. (1995). Intrathecal Amitriptyline Acts as an N-Methyl-D-Aspartate Receptor Antagonist in the Presence of Inflammatory Hyperalgesia in Rats. Anesthesiology 83 (5), 1046–1054. 10.1097/00000542-199511000-00018 7486155

[B17] Ellison G. (1995). The N-Methyl-D-Aspartate Antagonists Phencyclidine, Ketamine and Dizocilpine as Both Behavioral and Anatomical Models of the Dementias. Brain Res. Brain Res. Rev. 20 (2), 250–267. 10.1016/0165-0173(94)00014-g 7795658

[B18] Gillman P. K. (2007). Tricyclic Antidepressant Pharmacology and Therapeutic Drug Interactions Updated. Br. J. Pharmacol. 151 (6), 737–748. 10.1038/sj.bjp.0707253 17471183 PMC2014120

[B19] Glasgow N. G. Povysheva N. V. Azofeifa A. M. Johnson J. W. (2017). Memantine and Ketamine Differentially Alter NMDA Receptor Desensitization. J. Neurosci. 37 (40), 9686–9704. 10.1523/JNEUROSCI.1173-17.2017 28877967 PMC5628409

[B20] Han E. B. Stevens C. F. (2009). Development Regulates a Switch Between Post- and Presynaptic Strengthening in Response to Activity Deprivation. Proc. Natl. Acad. Sci. U S A. 106, 10817–10822. 10.1073/pnas.0903603106 19509338 PMC2705571

[B21] Heimstad E. Edvardsen O. Ferrin T. E. Dahl S. G. (1991). Molecular Structure and Dynamics of Tricyclic Antidepressant Drugs. Eur. Neuropsychopharmacol. 1 (2), 127–137. 10.1016/0924-977x(91)90714-6 1821702

[B22] Huang Y. Wen L. L. Xie J. D. Ouyang H. D. Chen D. T. Zeng W. A. (2019). Antinociceptive Effectiveness of the Inhibition of NCX Reverse-Mode Action in Rodent Neuropathic Pain Model. Mol. Pain 15, 1744806919864511. 10.1177/1744806919864511 31370728 PMC6681272

[B23] Iacobucci G. J. Popescu G. K. (2020). Ca2+-Dependent Inactivation of GluN2A and GluN2B NMDA Receptors Occurs by a Common Kinetic Mechanism. Biophys. J. 118 (4), 798–812. 10.1016/j.bpj.2019.07.057 31629478 PMC7036730

[B24] Kiss J. P. Szasz B. K. Fodor L. Mike A. Lenkey N. Kurkó D. (2012). GluN2B-containing NMDA Receptors as Possible Targets for the Neuroprotective and Antidepressant Effects of Fluoxetine. Neurochem. Int. 60 (2), 170–176. 10.1016/j.neuint.2011.12.005 22197911

[B25] Kurian R. Raza K. Shanthanna H. (2019). A Systematic Review and Meta-Analysis of Memantine for the Prevention or Treatment of Chronic Pain. Eur. J. Pain 23 (7), 1234–1250. 10.1002/ejp.1393 30848504

[B26] Lavoie P. A. Beauchamp G. Elie R. (1990). Tricyclic Antidepressants Inhibit Voltage-dependent Calcium Channels and Na(+)-Ca2+ Exchange in Rat Brain Cortex Synaptosomes. Can. J. Physiol. Pharmacol. 68, 1414–1418. 10.1139/y90-215 2285885

[B27] Lawson K. (2017). A Brief Review of the Pharmacology of Amitriptyline and Clinical Outcomes in Treating Fibromyalgia. Biomedicines 5, 24. 10.3390/biomedicines5020024 PMC5489810 28536367

[B28] Li L. Chen S. R. Chen H. Wen L. Hittelman W. N. Xie J. D. (2016). Chloride Homeostasis Critically Regulates Synaptic NMDA Receptor Activity in Neuropathic Pain. Cell Rep 15, 1376–1383. 10.1016/j.celrep.2016.04.039 27160909 PMC4871741

[B29] Maki B. A. Popescu G. K. (2014). Extracellular Ca(2+) Ions Reduce NMDA Receptor Conductance and Gating. J. Gen. Physiol. 144 (5), 379–392. 10.1085/jgp.201411244 25348411 PMC4210427

[B30] McTavish D. Benfield P. (1990). Clomipramine. An Overview of its Pharmacological Properties and a Review of its Therapeutic Use in Obsessive Compulsive Disorder and Panic Disorder. Drugs 39, 136–153. 10.2165/00003495-199039010-00010 2178909

[B31] Mironova E. V. Evstratova A. A. Antonov S. M. (2007). A Fluorescence Vital Assay for the Recognition and Quantification of Excitotoxic Cell Death by Necrosis and Apoptosis Using Confocal Microscopy on Neurons in Culture. J. Neurosci. Methods 163, 1–8. 10.1016/j.jneumeth.2007.02.010 17395268

[B32] Monyer H. Burnashev N. Laurie D. J. Sakmann B. Seeburg P. H. (1994). Developmental and Regional Expression in the Rat Brain and Functional Properties of Four NMDA Receptors. Neuron 12, 529–540. 10.1016/0896-6273(94)90210-0 7512349

[B33] Nothdurfter C. Tanasic S. Di Benedetto B. Uhr M. Wagner E. M. Gilling K. E. (2013). Lipid Raft Integrity Affects GABAA Receptor, but Not NMDA Receptor Modulation by Psychopharmacological Compounds. Int. J. Neuropsychopharmacol. 16, 1361–1371. 10.1017/S146114571200140X 23217923

[B34] Nowak L. Bregestovski P. Ascher P. Herbet A. Prochiantz A. (1984). Magnesium gates Glutamate-Activated Channels in Mouse Central Neurones. Nature 307 (5950), 462–465. 10.1038/307462a0 6320006

[B35] Pancrazio J. J. Kamatchi G. L. Roscoe A. K. Lynch C. (1998). Inhibition of Neuronal Na+ Channels by Antidepressant Drugs. J. Pharmacol. Exp. Ther. 284 (1), 208–214. 9435180

[B36] Paoletti P. Bellone C. Zhou Q. (2013). NMDA Receptor Subunit Diversity: Impact on Receptor Properties, Synaptic Plasticity and Disease. Nat. Rev. Neurosci. 14, 383–400. 10.1038/nrn3504 23686171

[B37] Peterson C. D. Kitto K. F. Verma H. Pflepsen K. Delpire E. Wilcox G. L. (2021). Agmatine Requires GluN2B-Containing NMDA Receptors to Inhibit the Development of Neuropathic Pain. Mol. Pain 17, 17448069211029171. 10.1177/17448069211029171 34210178 PMC8255568

[B38] Reynolds I. J. Miller R. J. (1988). Tricyclic Antidepressants Block N-Methyl-D-Aspartate Receptors: Similarities to the Action of Zinc. Br. J. Pharmacol. 95, 95–102. 10.1111/j.1476-5381.1988.tb16552.x 2905906 PMC1854115

[B39] Riediger C. Schuster T. Barlinn K. Maier S. Weitz J. Siepmann T. (2017). Adverse Effects of Antidepressants for Chronic Pain: A Systematic Review and Meta-Analysis. Front. Neurol. 8, 307. 10.3389/fneur.2017.00307 28769859 PMC5510574

[B40] Santos M. G. Tavares I. M. Barbosa A. F. Bettini J. Figueiredo E. C. (2017). Analysis of Tricyclic Antidepressants in Human Plasma Using Online-Restricted Access Molecularly Imprinted Solid Phase Extraction Followed by Direct Mass Spectrometry Identification/quantification. Talanta 163, 8–16. 10.1016/j.talanta.2016.10.047 27886774

[B41] Sernagor E. Kuhn D. Vyklicky L. Jr Mayer M. L. (1989). Open Channel Block of NMDA Receptor Responses Evoked by Tricyclic Antidepressants. Neuron 2 (3), 1221–1227. 10.1016/0896-6273(89)90306-1 2483111

[B42] Sessoms-Sikes S. Honse Y. Lovinger D. M. Colbran R. J. (2005). CaMKIIalpha Enhances the Desensitization of NR2B-Containing NMDA Receptors by an Autophosphorylation-dependent Mechanism. Mol. Cel. Neurosci. 29 (1), 139–147. 10.1016/j.mcn.2005.01.006 15866054

[B43] Shimizu T. Shibata M. Wakisaka S. Inoue T. Mashimo T. Yoshiya I. (2000). Intrathecal Lithium Reduces Neuropathic Pain Responses in a Rat Model of Peripheral Neuropathy. Pain 85 (1-2), 59–64. 10.1016/s0304-3959(99)00249-3 10692603

[B44] Sibarov D. A. Abushik P. A. Poguzhelskaya E. E. Bolshakov K. V. Antonov S. M. (2015). Inhibition of Plasma Membrane Na/Ca-Exchanger by KB-R7943 or Lithium Reveals its Role in Ca-dependent N-Methyl-D-Aspartate Receptor Inactivation. J. Pharmacol. Exp. Ther. 355, 484–495. 10.1124/jpet.115.227173 26391160

[B45] Sibarov D. A. Antonov S. M. (2018). Calcium-Dependent Desensitization of NMDA Receptors. Biochemistry (Mosc) 83, 1173–1183. 10.1134/S0006297918100036 30472955

[B46] Sibarov D. A. Poguzhelskaya E. E. Antonov S. M. (2018). Downregulation of Calcium-dependent NMDA Receptor Desensitization by Sodium-Calcium Exchangers: A Role of Membrane Cholesterol. BMC Neurosci. 19 (1), 73. 10.1186/s12868-018-0475-3 30419823 PMC6233507

[B47] Sobolevsky A. I. Koshelev S. G. Khodorov B. I. (1999). Probing of NMDA Channels with Fast Blockers. J. Neurosci. 19, 10611–10626. 10.1523/JNEUROSCI.19-24-10611.1999 10594045 PMC6784965

[B48] Stepanenko Y. D. Boikov S. I. Sibarov D. A. Abushik P. A. Vanchakova N. P. Belinskaia D. (2019). Dual Action of Amitriptyline on NMDA Receptors: Enhancement of Ca-dependent Desensitization and Trapping Channel Block. Sci. Rep. 9, 19454. 10.1038/s41598-019-56072-z 31857688 PMC6923474

[B49] Stern R. S. Marks I. M. Mawson D. Luscombe D. K. (1980). Clomipramine and Exposure for Compulsive Rituals: II. Plasma Levels, Side Effects and Outcome. Br. J. Psychiatry 136, 161–166. 10.1192/bjp.136.2.161 7370482

[B50] Tohda M. Urushihara H. Nomura Y. (1995). Inhibitory Effects of Antidepressants on NMDA-Induced Currents in Xenopus Oocytes Injected with Rat Brain RNA. Neurochem. Int. 26 (1), 53–58. 10.1016/0197-0186(94)00101-y 7787763

[B51] Vandel S. Bertschy G. Bonin B. Nezelof S. François T. H. Vandel B. (1992). Tricyclic Antidepressant Plasma Levels After Fluoxetine Addition. Neuropsychobiology 25 (4), 202–207. 10.1159/000118838 1454161

[B52] Vorobjev V. S. Sharonova I. N. (1994). Tetrahydroaminoacridine Blocks and Prolongs NMDA Receptor-Mediated Responses in a Voltage-dependent Manner. Eur. J. Pharmacol. 253, 1–8. 10.1016/0014-2999(94)90750-1 8013535

[B53] Watanabe J. Beck C. Kuner T. Premkumar L. S. Wollmuth L. P. (2002). DRPEER: A Motif in the Extracellular Vestibule Conferring High Ca2+ Flux Rates in NMDA Receptor Channels. J. Neurosci. 22 (23), 10209–10216. 10.1523/jneurosci.22-23-10209.2002 12451122 PMC6758750

[B54] Zahradník I. Minarovic I. Zahradníková A. (2008). Inhibition of the Cardiac L-type Calcium Channel Current by Antidepressant Drugs. J. Pharmacol. Exp. Ther. 324 (3), 977–984. 10.1124/jpet.107.132456 18048694

[B55] Zhang S. Ehlers M. D. Bernhardt J. P. Su C. T. Huganir R. L. (1998). Calmodulin Mediates Calcium-dependent Inactivation of N-Methyl-D-Aspartate Receptors. Neuron 21 (2), 443–453. 10.1016/s0896-6273(00)80553-x 9728925

